# Book Reviews

**Published:** 1910-09

**Authors:** 


### BOOK REVIEWS

THE NEW PSYCHOLOGY.—Its Basic Principle and Practical Formulas, by A. A. Lindsay, M. D. Published by Eugene and Arthur Lindsay, Portland, Oregon.

This book briefly describes the basis principles of psychology, phychotheraphy, how to treat disease and habits, hypnosis; how to produce and use, suggestion in moral reform, intelligence of the cells, and many other interesting phases of this subject.

CLINICAL STUDIES FOR NURSES.—By Charlotte A. Aikens, formerly Superintendent of Columbia Hospital, Pittsburg, etc. Illustrated. Pp. 510. W. B. Saunders & Co., Philadelphia.

This excellent work is well designed to assist second and third year pupil-nurses, as well as those engaged in caring for the sick, in procuring a systematic graded course of instruction. It is the second book of the series by the same author, the first being "Primary Studies for Nurses." Such books greatly simplify the teaching of nurses and at the same time provides a useful book for graduates. The careful manner in which it has been prepared makes commendation a necessity as well as a pleasure.

---

GYNECOLOGICAL DIAGNOSIS—By Walter L. Burrage, A. M., M. D. (Harv.) With two hundred and seven text illustrations. Pp. 656. D. Appleton & Co., New York.

In every branch of medicine great interest in the diagnosis has been taken in recent years and thus is medicine becoming more scientific and our results more uniform. It may be truly said that the patient is half cured when a *correct* diagnosis has been made. Burrage has given us a practical book embodying simplicity of technique and concise statement of essentials. The salient points of anatomy and the latest views of pathology have been summarized at the beginning of each chapter. The illustrations are excellent and an original feature is an alphabetical index of them which enables a reader to find quickly a desired cut.

---

DISEASES OF INFANCY AND CHILDHOOD—Their Dietetic, hygienic and medical treatment. A text-book designed for practitioners and students of medicine, by Louis Fisher, M. D., New York. Third edition, with 303 illustrations, several in color, and 29 full-page half-tone and color plates. Pp. 980. F. A. Davis & Co., Publishers, Philadelphia, Pa.

This well and favorably known work has been completely revised. Among the new things added a few may be mentioned:

The modern method of diagnosis and treatment of meningitis by the Flexner serum; infant feeding with emphasis upon the common sense part; the perhydrol method of preserving human milk; intravenous method of administering diphtheria antitixon; the hemostatic value of horse serum, etc., etc. All phases of infant feeding are thoroughly presented and a large amount of space devoted to this one but most important subject. The thoroughness and detail of the entire work make it a most reliable book.

---

A TEXT-BOOK OF PATHOLOGY. By Joseph McFarland, M. D., Professor of Pathology and Bacteriology in the Medico-Chirurgical College of Philadelphia, Second Edition. Octavo of 856 pages, with 437 illustrations, some in colors. Philadelphia and London—W. B. Saunders Company, 1910. Cloth $5.00 net; half-Morocco $6.50 net.

The autor has presented in a "succinct, intelligible and readable manner the facts every student should know before he advances from the elementary to the practical branches of medicine," and which the practitioner and surgeon should review sufficiently frequently to keep in mind a correct idea of the pathologic lesion they are seeking to cure.

McFarland stands high in the pathologic world and this work ranks among the best of its kind. It has been revised from cover to cover and advances which have appeared to have a genuine character have been added. Altogether it is a most readable and useful book.

---

SURGICAL AFTER-TREATMENT.—By L. R. G. Crandon, A. M., M. D., Assistant in Surgery at Harvard Medical School. Octavo of 803 pages, with 265 original illustrations. Philadelphia and London. W. B. Saunders Company, 1910. Cloth $6.00 net; Half Morocco $7.50 net.

Undoubtedly many surgical failures have been due to poor after-treatment, with equal certainty the reviewer asserts that a large percentage of the failures could have been prevented had the attendant been familiar with the facts contained in this treatise. The author states in the preface that the book was primarily written for two classes of practitioners; house surgeons in hospitals and general practitioners in communities which are not surgical centers; there is still another class who can obtain a large amount of useful information from this work and they are the surgeons who operate more or less infrequently and do not have a regular system of after-treatment as do the more prominent surgeons. The minute details, which often add so much to the comfort and well-being of the patient, are carefully elucidated in a common-sense and practical manner and is to be highly commended.

